# Molecular Dynamics Study on the Welding Behavior in Dissimilar TC4-TA17 Titanium Alloys

**DOI:** 10.3390/ma15165606

**Published:** 2022-08-16

**Authors:** Peng Ou, Zengqiang Cao, Ju Rong, Xiaohua Yu

**Affiliations:** 1 School of Mechanical Engineering, Northwestern Polytechnical University,127 Youyi Ave, West, Xi’an 710072, China; 2 Kunming Precision Machinery Research Institute, P.O. Box No.26, Kunming 650118, China; 3 Faculty of Materials Science and Engineering, Kunming University of Science and Technology, Kunming 650093, China

**Keywords:** molecular dynamics, TC4-TA17 alloy, radial distribution function, phase transition, diffusion coefficient

## Abstract

Titanium alloys have become the material of choice for marine parts manufacturing due to their high specific strength and excellent resistance to seawater corrosion. However, it is still challenging for a single titanium alloy to meet the comprehensive specifications of a structural component. In this study, we have applied a molecular dynamics approach to simulate the aging phase transformation, K-TIG welding process, and mechanical properties of the TC4-TA17 (Ti6Al4V-Ti4Al2V) alloy. The results show that during the aging phase transformation process, changes in the structure of the titanium alloys are mainly manifested in the precipitation of a new phase from the sub-stable β-phase, and after the state stabilization, the α-phase content reaches 45%. Moreover, during the melting and diffusion process of TC4-TA17, aluminum atoms near the interface diffuse, followed by titanium atoms, while relatively few vanadium atoms are involved in the diffusion. Finally, the results of tensile simulations of the TC4-TA17 alloy after welding showed that stress values can reach up to 9.07 GPa and that the mechanical properties of the alloy in the weld zone are better than those of the single alloys under the same conditions. This study will provide theoretical support for the optimization of process parameters for TC4-TA17 alloy welding.

## 1. Introduction

The exploration and exploitation of marine resources impose special requirements on seawater corrosion resistance along with the strength of engineering materials, and the selection of appropriate materials is crucial [1,2]. Titanium alloys have recently been demonstrated to be favorable materials for marine parts manufacturing due to their advantages of high specific strength and excellent seawater corrosion resistance [3,4]. However, it remains challenging for a single type of titanium alloy to satisfy the composite indicator of structural members in terms of performance and cost [5]. Specifically, considering a pressure-resistant hull of marine parts with bulged flanges, the flanges do not require as high a strength as the pressure-resistant hull because the pressures on either side of them are equivalent. Therefore, parts serving in the marine environment tend to require composite connections of dissimilar titanium alloys so as to achieve a property balance between the structures [6,7,8].

To fully exploit the performance characteristics of different types of titanium alloys and to reduce costs, it is necessary to adopt appropriate dissimilar titanium alloys for the pressure-resistant hull and flanges. The Ti6Al4V (TC4) titanium alloy displays great comprehensive performance, and it is suitable for various press-forming processes by virtue of its high strength and fine process plasticity, which is extensively applied in the pressure-resistant hulls of marine parts [9,10,11]. Ti4Al2V (TA17) belongs to a class of medium-strength titanium alloys with promising weldability, corrosion resistance, and impact toughness, and is particularly beneficial for the manufacture of loaded components, such as flanges and skeletons employed in the marine environment [12,13,14]. Actually, these two typical titanium alloys have shown great promise in marine parts manufacturing, but there is still a shortcoming regarding long-term service structural integrity when simply joining the two together. This effect is caused by an undesirable incompatibility at the connection interface owing to different features of TC4 and TA17. Based on the above analysis, some researchers consider that fusion welding (including K-TIG welding, electron beam welding, laser beam welding, and so on) with high precision, low distortion, and reliable connections offers encouraging potential [15,16]. Currently, it is difficult to investigate the narrow linker region of fusion welding from a macro-scale perspective, whereas the application of molecular dynamics to study the welding behavior of titanium alloys from a micro-scale perspective has been favored. Zhu et al. [17] examined the diffusion process of V atoms in titanium alloys, as a result of which the influence laws of V atom concentration on the diffusion distance and diffusion coefficient were obtained. Shimono et al. [18] performed molecular dynamics simulations of the recrystallization process of Ti–Al alloys in the amorphous state to derive the critical cooling rate of Ti–Al alloys; in addition, the way in which the Al content affects the amorphous formation ability of Ti–Al alloys was established. Semiatin et al. [19] experimentally probed the self-diffusion coefficients of each atom in TC4 alloy and expressed the Arrhenius function for the variation of diffusion coefficient with diffusion temperature. The studies mentioned above suggest that molecular dynamics is an effective simulation tool in material calculations. Nevertheless, most studies have focused on structural transformation processes in alloys and diffusion linkage processes between single-crystal dissimilar metals, whereas molecular dynamics simulations of diffusion linkage processes between dissimilar multi-alloy systems have been less well studied. Consequently, the aging transformation, fusion welding process, and mechanical properties of the TC4-TA17 (Ti6Al4V-Ti4Al2V) alloy need to be further investigated.

Here, a molecular dynamics study of K-TIG welding behavior between two ternary titanium alloys, TC4 and TA17, has been carried out. The initial configurations of TC4, TA17, and TC4-TA17, matching the corresponding atomic contents, were established by a random substitution method based on the LAMMPS (large-scale atomic/molecular massively parallel simulator) software package [20]. Precipitation of the α phase from the sub-stable β phase was simulated, and the stable α + β biphase structure was modeled by aging pretreatment of the TC4 configuration. On this basis, the effects of process parameters on the diffusion linker region width and Ti atom diffusion coefficients of TC4-TA17 dissimilar titanium alloys were evaluated. Furthermore, molecular dynamics simulations of tensile deformation behavior in the diffusion linker region were performed to acquire stress–strain curves with different process parameters. Molecular dynamics simulation results have revealed that TC4-TA17 exhibits satisfactory welding behavior in dissimilar titanium alloys. It is expected that this study will provide theoretical support for the optimization of process parameters for fusion welding and will further promote the practical application of dissimilar titanium alloys in marine parts manufacturing.

## 2. Computational Methods

### 2.1. Selection of Alloy Potential Function

A molecular dynamics approach was used to simulate the aging phase change behavior of the two Ti–Al–V ternary alloys and the welding melting process between them. In molecular dynamics, the state of motion of any particle can be obtained by integrating over Newton’s equations of motion [21], and the differential equation of Newton’s second law is as follows:

(1) $$m_{i}\frac{d^{2}r_{i}}{dt^{2}}=-\nabla_{i}V\left(r_{1},r_{2\cdots \cdots }r_{N}\right)$$

where *V*(*r*_1_, *r*_2_... *r*_N_) is the potential function of the atom, and $m_{i}\;\mathrm{and}\;r_{i}$ are the mass and position of the *i*-th atom, respectively. By solving for the state quantities (velocity and position) of the particles, the trajectory of the system can be described. By averaging the results for the system using statistical methods, the required macroscopic physical quantities, such as temperature, pressure, stress, etc., can be obtained to investigate the equilibrium thermodynamic properties of the system and the microstructure of the particles.

In molecular dynamics simulations, the interaction potential between atoms is defined by a mathematical function of atomic potential energy versus coordinates [21,22]. The embedded atom method (EAM) is a semi-empirical theory, proposed by Daw and Baskes in 1983, based on density generalized theory using effective medium and quasi-atomic approximations [23,24,25,26]. EAM theory treats each atom in a system as an exotic impurity of the substrate under study, and the system energy is expressed as the sum of the embedding energy and the interaction potential energy, so that polyatomic interactions can be included in the embedding energy. EAM is applicable to metallic atomic systems and provides a better description of the way in which bonds are formed between atoms compared to other potential functions. The potential function is generalized from the two-body potential to take into account the free electron gas effect in the local region of each atom, using the gel model to simplify the description of the complex environment surrounding the atom. Its functional form is as follows [24]:

(2) $$U_{total}=\sum_{i}^{}F\left(p_{i}\right)+\frac{1}{2}\sum_{i}^{}\sum_{j\neq i}^{}\emptyset \left(r_{ij}\right)$$

(3) $$p_{i}=\sum_{j\neq i}^{}f\left(r_{ij}\right)$$

where F is the atomic embedding energy as a function of $p_{i}$, ∅ is the short-range pair potential, and $p_{i}$ is a function of atomic electron density. The first term on the right-hand side of Equation (2) is the embedding energy, which represents the energy at which the atom is embedded at the electron density of the substrate, and the second term is the conventional pair potential, which describes the repulsion between atoms in a solid system. The EAM model parameters are determined by fitting the binding energy, single vacancy formation energy, lattice constant, elastic constant, and structural energy difference of the elements. From Equations (2) and (3), the force between atoms can be deduced as:

(4) $$F_{ij}=-\{\left[F^{\prime }\left(p_{i}\right)+F^{\prime }\left(p_{j}\right)\right]f^{\prime }\left(r_{ij}\right)+\varphi^{\prime }\left(r_{ij}\right)\}$$

Zhou and co-workers developed a library of alloy potentials for 16 commonly used metal elements, including Ti, Al, and Cu, and were able to derive alloy potential function files for any combination through a fitting procedure, but the alloy library did not contain the element V [25]. In 2021, Zhao et al. added V to the existing alloy library [25]. Based on the above work, a fitting procedure is applied here to generate the required Ti–Al–V alloy potential function files. Under the above conditions, the Nosé–Hover temperature control method was used to simulate the molecular dynamics of the welding melting process of TC4 and TA17 alloys. The calculated melting point of TC4 alloy is about 1780 K, as compared to the actual melting point of about 1950 K. The simulated value is close to the actual value, which shows the suitability of the selected potential function.

### 2.2. The Establishment of an Atomic Model

**Figure 1:**
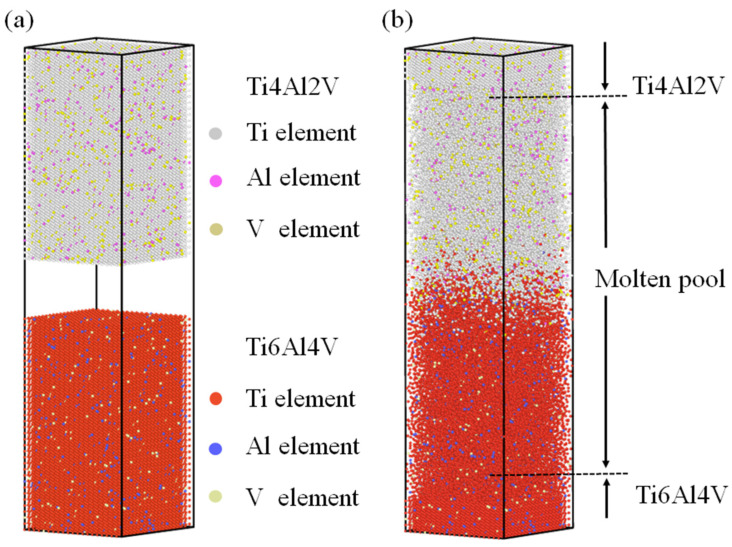
Welding model of TC4-TA17 dissimilar titanium alloy: (**a**) molecular dynamics calculation model before welding, in which the upper part is TC4 titanium alloy and the lower part is TA17 alloy; (**b**) molecular dynamics structure after welding, in which the middle is the molten pool area. The alloys form an obvious metallurgical bond.

The initial three-dimensional configurations of the two alloys were set as bcc structures, and the system in which they were located used NVT system synthesis. Considering the scale effect, periodic boundary conditions were set in the *x* and *y* directions, and contractive boundary conditions were set in the *z* direction. The box size was set to 20 × 20 × 80 (bcc structured cubic cell, Ti lattice constant 0.328 nm), with the TC4 alloy occupying the lower part of the box with a size of 20 × 20 × 35 and the TA17 alloy occupying the upper part of the box with the same dimensions. The total number of atoms was 56,800. In the TC4 alloy model, corresponding Ti atoms were replaced with 6 at% Al atoms and 4 at% V atoms at random, whereas in the TA17 alloy model, the corresponding Ti atoms were replaced with 4 at% Al atoms and 2 at% V atoms at random. The resulting initial atomic configuration is shown in Figure 1. For ease of observation, the configurations above and below the contact surface are distinguished by different colors. The Ti atoms are colored gray and red, the Al atoms are colored pink and blue, and the V atoms are colored brown and light green.

### 2.3. Phase Change, Welding and Stretching Process Simulation

Having established the atomic model, the phase change simulation process was first carried out with LAMMPS to obtain the initial state of the material. This was followed by a simulation of the melting and welding process, and finally, the tensile mechanical properties of the alloy were tested. All simulation parameter settings are basically the same as in previous references [17,18,19] to ensure that the simulation can be repeated.

The phase change temperature of the general α + β titanium alloy was 900 K. The sub-stable phase formed by high-temperature quenching of the general α + β titanium alloy was followed by precipitation of a new phase as a result of a phase change process in the lower phase temperature region, significantly strengthening the alloy. The phase synthesis was run for 6000 steps, and atomic information was recorded at intervals of 20 steps. In a simulation of the aging treatment of the TC4 and TA17 alloys, the three atomic boundary layers above and below the model were fixed and stiffened, and a z-directional load was applied to each atom in this boundary layer, similar to rigid die pressurization, so that a uniform pressure was applied. The simulation step was set to 0.01 ps (1 ps = 10–12 s).

Before simulating the welding melting process, the initial temperature of the model was set to 1200 K. After running 80,000 steps, the temperature was raised to 2000 K to melt the alloy. The model was then held at 2000 K for 100 ps to obtain the diffusion of each atom throughout the melting and diffusion processes.

Finally, molecular dynamics simulations were used to obtain the changes in the mechanical properties of the alloy before and after melting, as well as its stress–strain curves at different temperatures and after different holding times. In order to better repeat our simulation, the detailed input file can be downloaded from the supporting materials.

## 3. Results and Discussion

### 3.1. Simulation of the Aging Phase Transition

**Figure 2:**
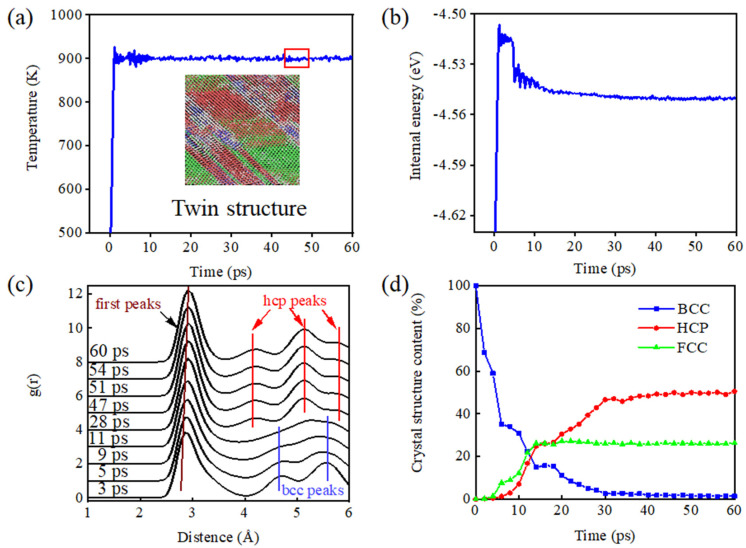
Microstructure of TC4 alloy after heat treatment and return to room temperature: (**a**) the relaxation process of the transformation from high-temperature face-centered cubic phase to low-temperature close-packed hexagonal phase finally forms a partial twin structure; (**b**) the change in binding energy during phase transformation; (**c**) the radial distribution function during phase transformation; and (**d**) analysis of the relative contents of each phase.

The microscopic details associated with atoms can be captured more intuitively by molecular dynamics simulation methods. Figure 2a shows a schematic diagram of the dot arrangement on the (100) crystal plane after completion of the aging phase transformation of the TC4 (as an example; the TA17 alloy has similar properties) primary protocell model. From the figure, it can easily be seen that the TC4 protocell model shows a twin structure after the precipitation phase, as well as some regions of stacking layer dislocations, where the red region is the hcp structure in the form of lamellae and the green region is the fcc structure in the form of stripes and small lamellae. Pinsook et al. [27] also observed striped fcc structures in their simulations of the bcc–hcp martensite phase transformation process of Zr and attributed this phenomenon to the fact that phase transformation stresses changed the stacking order of the hcp structure, resulting in the formation of fcc structures in some regions, and that the formation of stacking layer dislocations and twin boundaries could reduce some of the stresses. In addition, the structure of multiple atomic layers may also be formed as transition regions at the intersection of multiple grains of different orientations.

Figure 2b shows the trend of the internal energy of the Ti–Al alloy system during the simulation. It can easily be seen that the internal energy shows a trend of significant decrease with increasing simulation time, followed by a slow decrease until it reaches a plateau. This is due to the fact that the β-phase of the Ti–Al alloy is sub-stable under the set simulated temperature conditions, and shifts to the thermodynamically more stable α-phase when aging at this temperature to reduce the system energy. In molecular dynamics simulations, the radial distribution function (RDF) is commonly used to characterize the degree of ordering of the dotted structure [28,29,30]. Figure 2c shows RDF plots of the TC4 ternary alloy at different moments during the aging phase transformation. As can be seen in the figure, the first peak of each plot incrementally shifts to the right with time, and its width increases. This change is due to the fact that the atoms of the bcc structure have eight nearest-neighbor atoms at 0.284 nm and six next-nearest-neighbor atoms at 0.322 nm. It is also evident from Figure 2c that the wave crest becomes wider due to the atomic vibrational shift. However, as the model undergoes a phase transition and the atoms are relatively displaced, the intensity of the characteristic peak corresponding to the initial bcc structure gradually decreases and the peak profile gradually becomes flat. The bcc structure gradually becomes an hcp structure with 12 nearest-neighbor atoms, the position of the first peak of the RDF curve is shifted to 0.295 nm, and the height and width of the peak increase due to the increase in coordination number. TC4 shows a shift from the characteristic peak of the bcc structure to the characteristic peak of the hcp structure at 11 ps.

Figure 2d shows plots of the relative contents of the bcc and hcp structures and the fcc structure versus simulation time. It can be seen from the figure that, during the first 14 ps, the model bcc structure content decreased sharply and the hcp and fcc structure contents gradually increased. The rates of change of the bcc and hcp structure contents slowed down during the period 15–30 ps, while the fcc structure content stabilized after 15 ps. After 30 ps, the contents of the crystal structures all stabilized, with the hcp structure content at about 50%, the fcc structure content at about 27%, and the bcc structure content close to 0.

**Figure 3:**
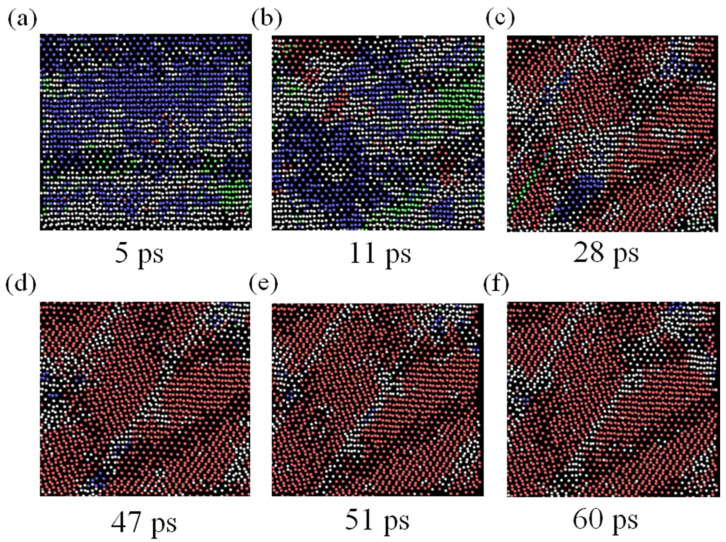
Structural evolution of TC4 alloy from high-temperature phase to room-temperature phase: (**a**–**f**) the corresponding structures at 5 ps, 11 ps, 28 ps, 47 ps, 51 ps, and 60 ps, respectively.

For a better visualization of changes in the crystal structure during phase transformation of the model, the co-nearest-neighbor angle distribution analysis method proposed by Ackland et al. [30] was used for processing, and then the phase transformation of the TC4 alloy was visualized with the aid of OVITO software [31,32]. Figure 3 depicts the evolution of the atomic dot structure of the (011) crystal plane with simulation time. The blue atomic region in the figure shows the bcc dot structure, the red atomic region shows the hcp dot structure, and the green atomic region shows the fcc dot structure. As can be seen from the figure, by 5 ps, most of the atoms in the model had deviated from the structural dot matrix due to the displacement generated by vibrations; by 11 ps, hcp atomic clusters started to form in the crystal structure and the new phase started to nucleate. This result is consistent with the plateau period in Figure 2c. As shown in Figure 3c, the new phase nucleated at the very beginning increased significantly, indicating that the process of new phase growth mainly occurred in the late stage of the TC4 aging phase transformation process; by 47 ps, the crystal structure had stabilized.

### 3.2. Simulation of K-TIG Welding of TC4-TA17 Alloy

**Figure 4:**
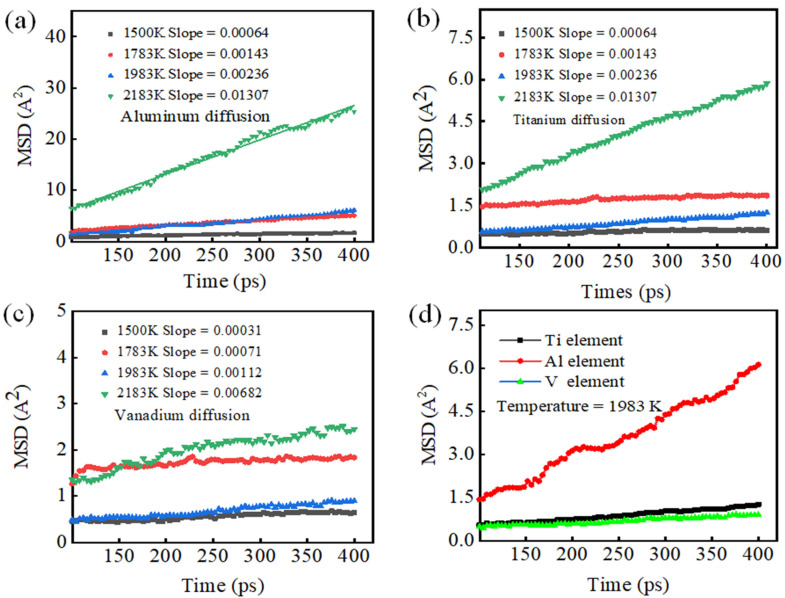
Diffusion mechanism of Al, Ti, and V atoms during welding: (**a**–**c**) root-mean-square displacements of Al, Ti, and V atoms at different temperatures; (**d**) root-mean-square displacements of Al, Ti, and V atoms at 1983 K.

Figure 4 shows distribution plots of root-mean-square deviation (RMSD) with holding time for Ti, Al, and V at holding temperatures of 1500–2183 K. In Figure 4a–c, the RMSDs for the Ti, Al, and V atoms show linear variations with holding time. The magnitude of the RMSD gradually increases with increasing holding temperature. This indicates that the higher the temperature, the higher the frequency of Ti, Al, and V atoms jumping in the connection interface [33,34,35,36]. Atomic diffusion coefficients were calculated by applying the mean-square difference formula. Mean-square displacement yielded diffusion coefficients of the corresponding elements, with a slope of the simulation time distribution plot of 1/6 [32]. Linear fitting of the RMSD data was carried out using Origin software. When the holding temperature was below 1983 K, the slopes for the Ti, Al, and V atoms were smaller. This implies that their atomic diffusion coefficients were small. When the holding temperature was higher than 1983 K, sharp increases in the slopes of the diffusion coefficients were observed, implying larger atomic diffusion coefficients. From Figure 4d, it can be seen that at the same holding temperature, Ti and V atomic diffusion coefficients are comparable in magnitude, whereas that of Al is larger.

**Figure 5:**
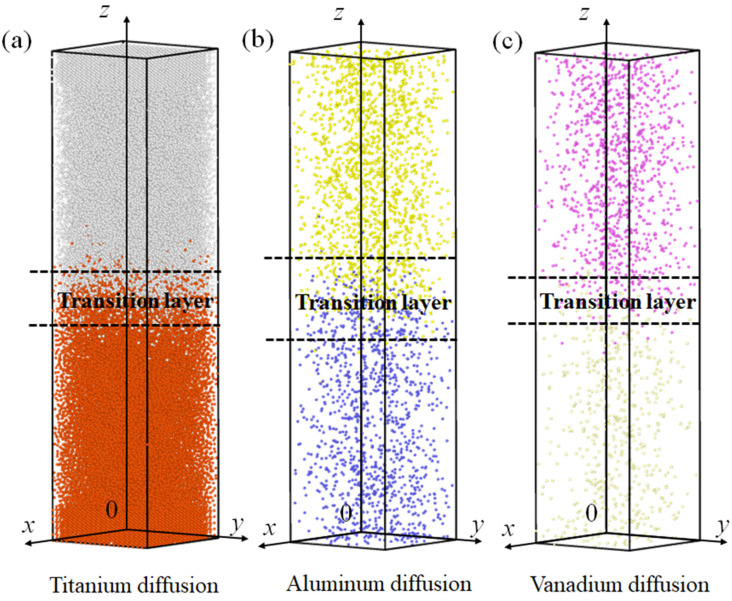
Analysis of the TC4-TA17 dissimilar titanium alloy welding diffusion layer: (**a**–**c**) diffusion layer structures of Al, Ti, and V atoms, respectively.

Figure 5 shows the atomic distributions of Ti, Al, and V with the holding time and pressure fixed at 400 ps and 3.5 MPa, respectively, at a melting temperature of 1983 K after complete diffusion. For ease of observation, the Ti, Al, and V atoms of the TA17 model above the contact surface are colored gray, yellow, and purple, respectively. The Ti, Al, and V atoms of the TC4 model below the contact surface are colored red, blue, and light yellow, respectively. It can be seen that at constant holding time and pressure, when the system temperature is 1983 K, it is mainly the Al atoms near the interface that diffuse, followed by the Ti atoms, whilst the V atoms have the lowest diffusion capacity; this is consistent with the results in Figure 4d [37,38].

**Figure 6:**
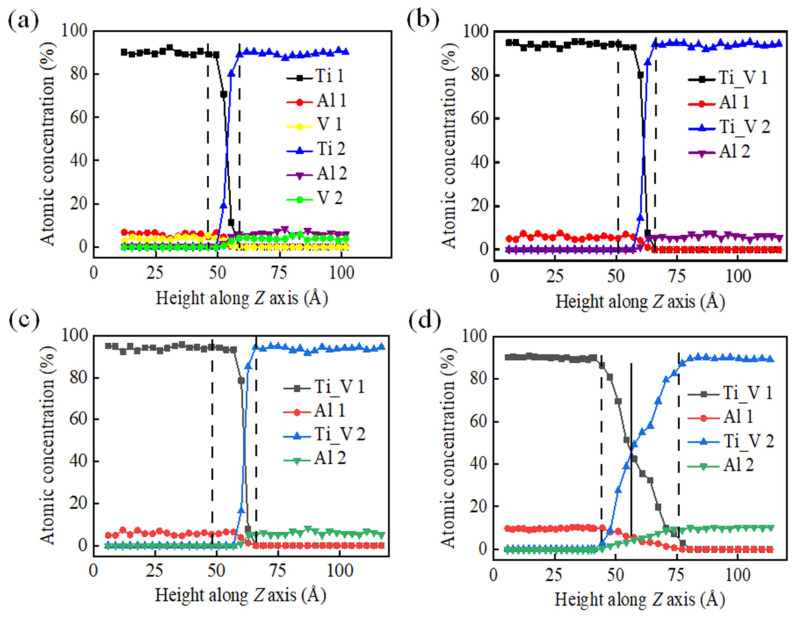
Fine structures of diffusion layers at different holding temperatures, as calculated from atomic positions using MATLAB software: (**a**–**d**) 1000 K, 1200 K, 1400 K, and 1600 K, respectively.

The main factors affecting the results of TC4-TA17 alloy diffusion bonding are holding temperature, pressure, holding time, and cooling rate. We proceeded by examining the effect of holding temperature on the diffusion bonding process of the TC4-TA17 alloy. Figure 6 shows the distributions of Ti–Al–V atomic concentrations at different holding temperatures. In the figure, Ti_V1 and Al1 are the atoms of the model below the contact surface, and Ti_V2 and Al2 are the atoms of the model above the contact surface. Since the initial content of V atoms was not high and the content in the diffusion region was even lower, to facilitate calculation processing and observation, we summed the atomic concentrations of Ti_V and did not calculate them separately. The areas between the dashed lines in Figure 6 represent the diffusion transition layers. It is generally stipulated that the area in which each atom concentration exceeds 5% in the contact surface and the lower part is the transition layer, so that the value of the diffusion connection width can be determined. It can be seen from the figure that as the heat preservation temperature was increased, the width of the diffusion connection increased significantly. A higher holding temperature can promote plastic yielding of the diffusion bonding interface, thereby expanding the contact area and reducing the probability of voids.

### 3.3. Simulation of Tensile Mechanical Properties

**Figure 7:**
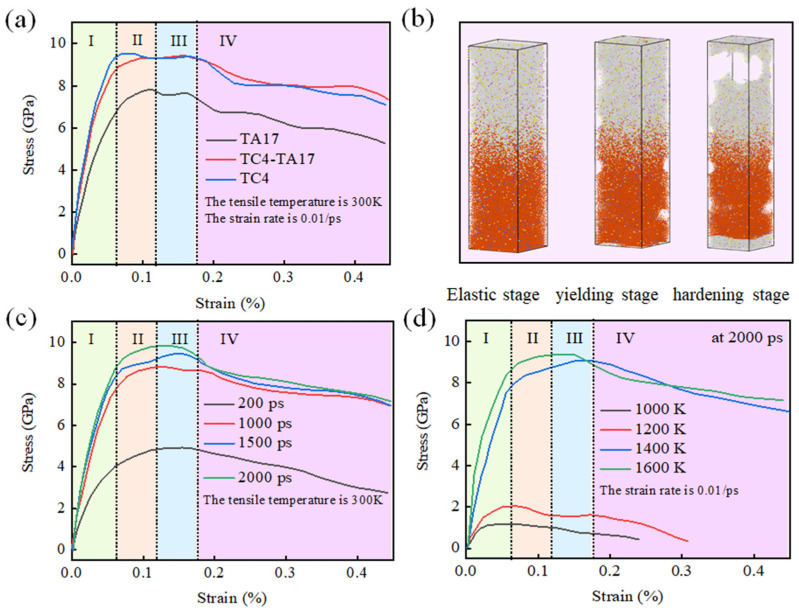
Fracture characteristics and fracture mechanism analysis of TC4-TA17 dissimilar titanium alloy after welding: (**a**) fracture characteristics of TC4-TA17 dissimilar titanium alloy at the tensile temperature is 300 K and the strain rate is 0.01/ps; and (**b**) fracture mechanism; (**c**) strain rate (the tensile temperature of 300 K); and (**d**) effect of holding temperature on tensile properties (the strain rate is 0.01/ps).

Figure 7 shows tensile stress–strain curves for TC4-TA17 with different parameters. As can be seen in Figure 7a, stress in TC4-TA17 increases with strain in stage I (ε = 0–0.07) and in stage II (ε = 0.05–0.12), similar stages to those of elastic deformation in the macroscopic materials. In stage III (ε = 0.12–0.18), a peak stress value of 9.07 GPa is reached at ε = 0.16 [39]. Thereafter, the stress decreases due to the continuous fracture of metal bonds; at the same time, the atoms are displaced by plastic deformation and then form new bonds with other metal atoms, causing the stress to increase. In stage IV (ε > 0.18), with increasing strain, the rate of metal bond breakage exceeds the rate of formation of new metal bonds and the stress begins to decrease; the TC4-TA17 shows the onset of necking and eventually fractures. In Figure 7b, with the increase in tensile force, the model of the upper and lower sides of the atomic movement of different degrees reaches tensile fracture, while the welding position is relatively solid. This indicates that the tensile mechanical properties of the TA17 and TC4 welding areas are good, exceeding those of TA17 and TC4 individually. It is worth noting that the tensile curve and fracture locations calculated by us are basically consistent with the experimental results, which proves that the simulation results are satisfactory [40,41,42,43,44].

As shown in Figure 7c, we simulated tensile experiments of TC4-TA17 welding samples under different holding times. It was found that as the holding time was increased, the initial elastic phase of the TC4-TA17 welded samples was prolonged and the tensile strength was increased. As can be seen in Figure 7d, with the increase in holding temperature, the yield strength of the TC4-TA17 welded samples also increased. At holding temperatures of 1000 K and 1200 K, the elastic phase was short, the yield point was at ε = 0.07, and the yield strengths were low. At holding temperatures of 1400 K and 1600 K, there was a significant extension of the resilience phase, the yield point appeared at ε = 0.15, and the peak yield strength was significantly higher than those at 1000 K and 1200 K. This indicates that the tensile mechanical properties of the TC4-TA17 welded samples are significantly improved with the increase in holding temperature.

## 4. Conclusions

(1) During the phase transformation process, crystal defects such as lamination and twin boundary are prone to occurring at the junction of grain boundaries, so as to alleviate stress caused by the distortion. Increasing the content of Al, as an α-phase-stabilizing element, facilitates nucleation and precipitation of this phase and facilitates the phase change.
(2) A higher holding temperature promotes the plastic yield of the diffusion connection interface, thus enlarging the contact area and reducing the probability of void formation.
(3) The tensile process in TC4-TA17 simulation is similar to those of macroscopic materials, involving elastic deformation and a plastic deformation stage, and showing a necking phenomenon. When the system enters plastic deformation, the stress–strain curve shows a spike, and high yield strength is acquired.

## Data Availability

The data that support the findings of this study are available upon reasonable request from the authors.
